# Supplementary material for: Scalable high performance radio frequency electronics based on large domain bilayer MoS2
Source: Nat Commun. 2018 Nov 14;9:4778. doi: 10.1038/s41467-018-07135-8 (PMC6235828; doi:10.1038/s41467-018-07135-8)
Supplement: Supplementary file 1 — Supplementary Information [file 41467_2018_7135_MOESM1_ESM.pdf]

Supplementary Information

Scalable high performance radio frequency electronics based on large domain bilayer MoS_2

Qingguo Gao [‡], Zhenfeng Zhang [‡], Xiaole Xu, Jian Song, Xuefei Li and Yanqing Wu[*]

Wuhan National High Magnetic Field Center and School of Optical and Electronic Information, Huazhong University of Science and Technology, Wuhan 430074, China

[‡]These authors contributed equally.

[*]Correspondence and requests for materials should be addressed to Y.W. (email: yqwu@mail.hust.edu.cn)

Supplementary Note 1. Discussion on the growth mechanism of large domain bilayer MoS_2.

To explain the reaction mechanism of domain size increased with MoO_3 weight increasing, the mass-driving kinetic model of MoS_2 CVD growth was constructed as Supplementary Figure 1, referring to the graphene and TMDCs growth model by Bhaviripudi et al and Zhou et al, respectively[1, 2]. Supplementary Figure 1a presents a schematic diagram of the kinetic model of MoS_2 film synthesis by CVD. A boundary layer is defined for a steady state between bulk gas flow region and substrate surface region, in which the gas flow is stagnant. A general chemical reaction equation for CVD can be as follows:

$2MoO_3$ (g) + $7S$ (g) → $2MoS_2 + 3SO_2$ (g) (Supplementary Equations 1)

First, the vaporized precursors MoO_3 and S are transported by the carrier gas, or diffused for the concentration gradient on the top of the substrate, and then diffuse through the boundary layer and get adsorbed on the surface. Vaporized MoO_3 and S react with each other and form the product MoS_2 at 1103 K, while another product SO_2 diffuse away from the surface through the boundary layer and is expelled away by the bulk gas flow. The reaction process above-described can be classified into two regime: the mass transport region, controlled by the diffusion rate of precursor through the boundary layer and the diffusion out through this layer of the gaseous products[3], and the surface reaction region, mainly depending on the substrate temperature. The equations for these two fluxes are given by:

$F_{\text{mass-transport}} = h_g (C_g - C_s)$ (Supplementary Equations 2)

$F_{\text{surface-reaction}} = K_s C_s$ (Supplementary Equations 3)

Where the $F_{\text{mass-transport}}$ is the flux of the active species through the boundary layer, $F_{\text{surface-reaction}}$ is the flux of consumed active species at the surface, h_g is the mass transport coefficient, K_s is the surface reaction constant, C_g is the concentration of gas in the bulk, and C_s is the concentration of active species at the surface. These two fluxes are successive, hence the slower process is the rate-limiting step during the MoS_2 synthesis. At steady state, $F_{\text{mass-transport}} = F_{\text{surface-reaction}} = F_{\text{total-flux}}$, and after eliminating C_s, $F_{\text{total-flux}}$ can be rewritten as:

$[K_s h_g / (K_s + h_g)]\, C_g$ (Supplementary Equations 4)

Mathematically, three regimes appear: $h_g >> K_s$ (surface reaction controlled region), $h_g \sim K_s$ (mixed region), and $h_g << K_s$ (mass transport limited region). At high temperatures, under typical APCVD

conditions, mass transport through the boundary layer is rate limiting ($h_g << K_s$).

We assume the sulfur vapor is sufficient for the reaction. Only the MoO_3 concentration is discussed in our mass-driving kinetic model. Four regimes are discussed here, distinguished by the weight of MoO_3 powder. In Supplementary Figure 1a, ~1 mg MoO_3 exhibit a monolayer single domain growth. We classify this self-limiting growth corresponding to the low mass flux. With the MoO_3 weight increased above ~1.5 mg, in Supplementary Figure 1b, the self-limiting growth break down and MoS_2 bilayer domains appear. We classify the breakdown of self-limiting growth corresponding to the high mass flux, which can provide enough source for large size nuclei for the layer-by-layer growth[4]. At high reaction temperature (1103 K), the surface reaction rate take place much faster according to the Arrhenius term, compared with the mass transport rate ($h_g << K_s$). Located in the mass transport limited region, higher precursor mass flux can contribute to higher diffusion rate through boundary layer, resulting in overcoming the mass transport limit and promoting single domain growth. When the weight of MoO_3 increased to 6 mg, in Supplementary Figure 1c, more precursor diffuse through the boundary layer and react on the surface ($h_g \sim K_s$). In this mixed region, domain growth is no longer restricted by one part, and the largest single domain can be obtain in this region. Moreover, trilayer or thicker MoS_2 domains appear for the high mass flux, however, only bilayer is discussed here. When the weight of MoO_3 reached 8 mg, in Supplementary Figure 1d, the single domain size of bilayer decreased and thick particulates appear. We attribute the decreasing domain size of bilayer to the excess mass flux diffuse into the boundary layer and hinder the gas product (SO_2) escape from the boundary layer. Moreover, the excess mass flux can possibly lead to gas phase reactions in the bulk gas flow, resulting to the MoS_2 particulates deposited on the substrate. The domain size depend on MoO_3 weight is depicted in Supplementary Figure 2.

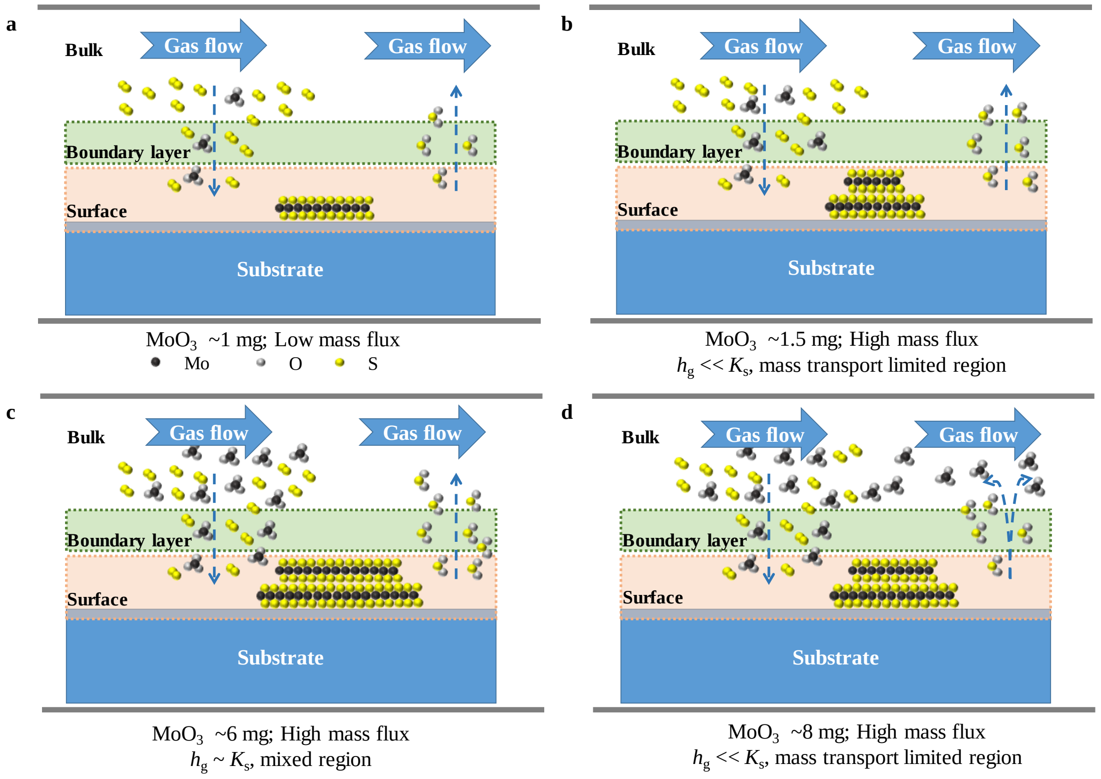

Supplementary Figure 1. Mass-driving kinetic model. **a,** Low mass flux region (MoO_3 ~1 mg). High mass flux (MoO_3 > 1 mg) at **b,** mass transport limited region, **c,** mixed region and d. mass transport limited region again.

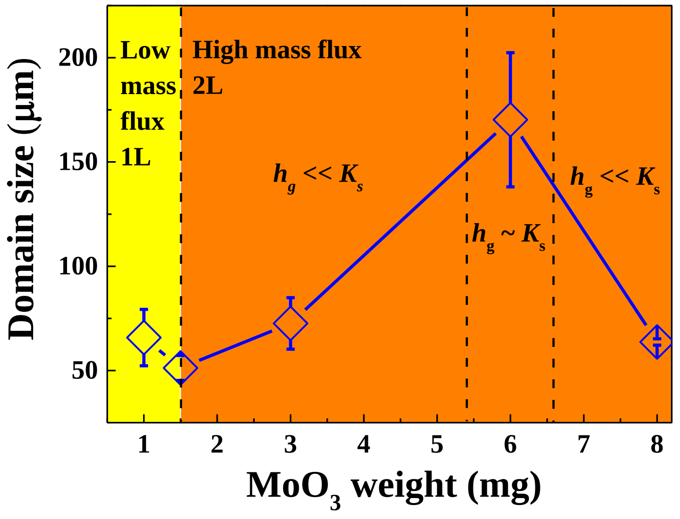

Supplementary Figure 2. The curve of relationship between domain sizes with MoO_3 weight. The error bar indicates the standard deviation. The domain sizes was collected from 55 flakes in 5 different growth temperature samples.

Supplementary Note 2. Discussion on the role of molten glass substrate

Molten glass (soda-lime silica) was used here for providing a “liquid-state” like surface at growth temperature 1103 K. On the one hand, different from conventional rigid substrate (thermal SiO_2/Si, sapphire etc.), the melting and regeneration of molten glass generate a smooth surface with ultralow defect density. Due to the nucleation of crystallites occurs preferentially at impurities or defect sites, nucleation density will drastically reduce with the decrease of defect sites, thus newly-generated nucleus mainly contribute to the growth of the MoS_2 films rather than forming new nucleation, which promoting the growth of large single-crystal[5]. On the other hand, molten glass lowers the migration barrier energy (U) of adatoms. The migration coefficient D is related to U by

$$D \approx D_{\infty} \exp(-U/k_B T) \quad \text{(Supplementary Equations 5)}$$

Where k_B is the Boltzmann constant and T is the temperature[6]. The migration coefficient on molten glass is much higher than that on solid substrates, which contributes to a high growth rate. Moreover, Yang et al had conducted experiments and confirmed that Na element in the soda-lime glass is considered to serve as an intermediate catalyst in the rapid growth of MoS_2[7].

Besides, because of the hydrophobicity/hydrophilicity of the MoS_2/glass substrate, MoS_2 film can be separated from glass immediately by immersing in deionized water. This short-time and safe transfer method make MoS_2 film free from structural damage and performance degradation. Furthermore, PMMA, gold or other films can be coated or deposited on MoS_2/glass stack as a supporting layer if necessary for large area transfer or contaminant-free contact.

Overall, molten glass plays an important role in promoting MoS_2 large domain growth and etching-free, short-time transfer.

Supplementary Note 3. Optical and SEM images of bilayer MoS_2 domains with different weight of MoO_3.

As shown in Supplementary Figure 3, monolayer MoS_2 domain size of ~50 - 82 μm are obtained when the MoO_3 weight equal to 1 mg. With the MoO_3 weight increasing to 1.5 and 3 mg, bilayer MoS_2 appear with domain size of 44 - 53 μm and 61 - 90 μm, respectively. Further increase the weight of MoO_3 powder to 6 mg, the bilayer domain size significantly increase up to 125 - 200 μm. When the MoO_3 weight exceed 6 mg, the bilayer single-crystal size begins to decrease and thicker nucleation sites appear. According to the optical images of concentric triangular geometry, AA stacked order of these two layers is confirmed[8, 9]. Supplementary Figure 4 shows the SEM images in various areas.

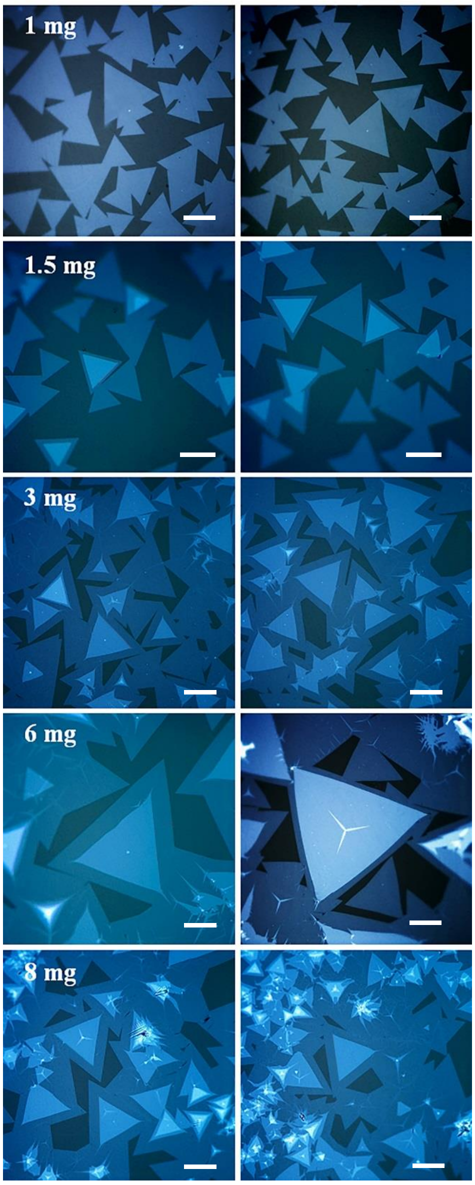

Supplementary Figure 3. Optical images of bilayer MoS_2 with varied MoO_3 weight. All the scale bars are 40 μm.

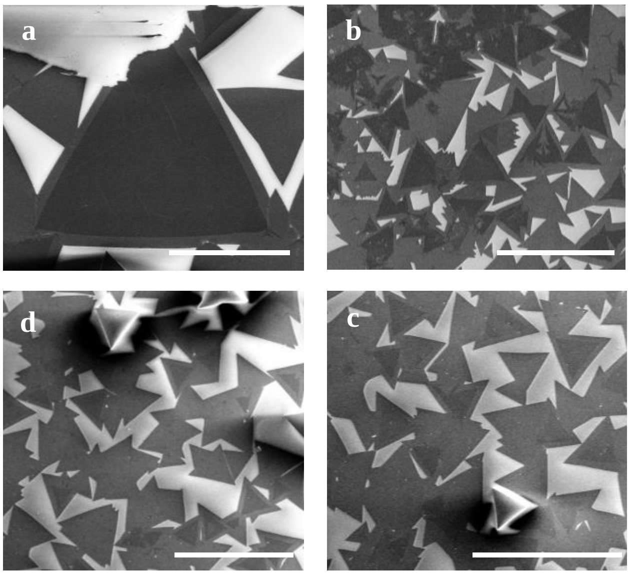

Supplementary Figure 4. SEM images of growth bilayer MoS_2 for varied MoO_3 weight. **a, b,** for 6 mg and **c, d,** for 1.5 mg. Scale bars are 100, 400, 200, 200 μm in a-d, respectively.

Supplementary Note 4. TEM characterizations

Supplementary Figure 5 shows the STEM images of the bilayer boundaries. Supplementary Figure 6 shows the high resolution TEM images of the bilayer MoS2 and the diffraction patterns. Supplementary Figure 7 shows the detailed Selected area electron diffraction (SEAD) patterns.

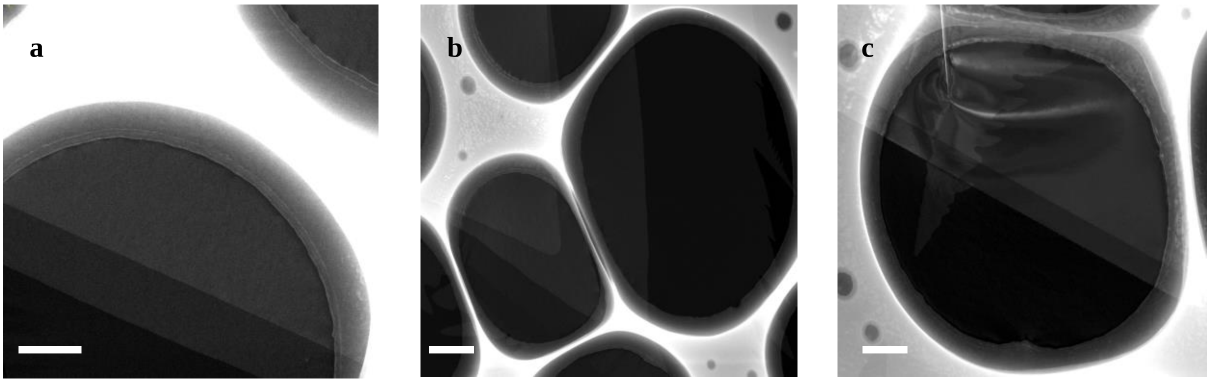

Supplementary Figure 5. Low resolution dark field STEM images of few layer MoS_2 boundary on carbon-coated micro copper grid. Accelerating voltage is 200 kV. Scale bars are 500 nm, 1 μm, 500 nm in a-c, respectively.

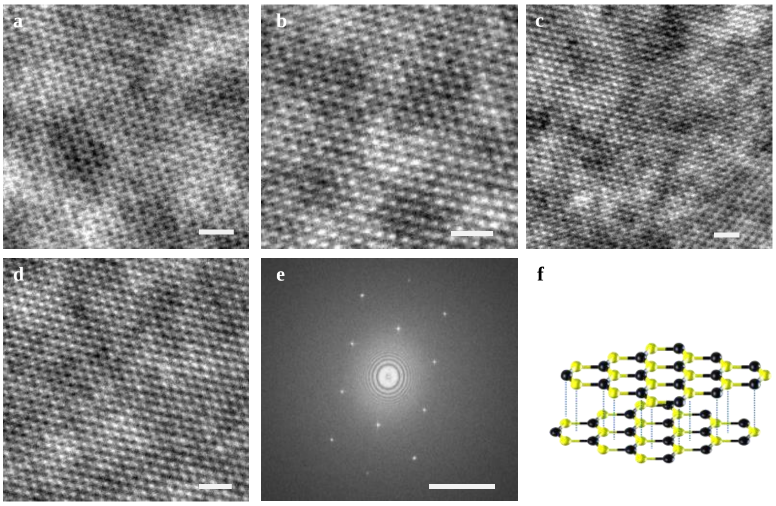

Supplementary Figure 6. TEM characterizations of MoS_2 bilayer. **a-d**, HRTEM images of AA stacked bilayer MoS_2 on carbon film TEM grid. All of them the scale bars are 1 nm. **e**, FFT image of MoS_2 bilayer exhibit only one set of hexagonally arranged diffraction spots, indicating the single crystalline nature of the flake over a large area and almost 0° twisted angle between first- and second-layers[8]. Scale bar, 5 nm^{-1}. **f**, Atomic configuration of AA stacked MoS_2 bilayer. AA stacked order of these two layer is confirmed, where S atoms of the top layer overlapped with the hexagonal centers of the bottom layer.

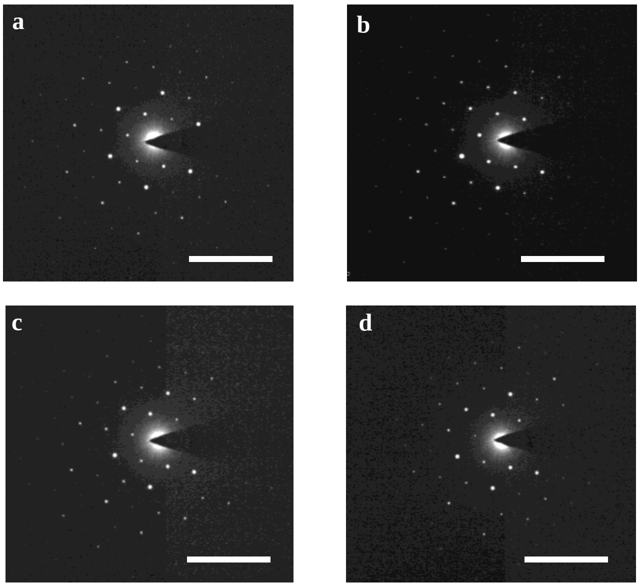

Supplementary Figure 7. **a-d,** Selected area electron diffraction (SAED) patterns taken from different holes of the TEM grid labeled “2-5” in Figure 2f. The near identical orientations of the diffraction patterns across these spatially diverse regions confirm that the bilayer MoS_2 domain is single crystalline. Scale bars are 5 nm^{-1}.

Supplementary Note 5. Transfer method of bilayer MoS_2 grown on molten glass.

As shown in Supplementary Figure 8, bilayer MoS_2 was transferred on HfLaO/Si or polyimide substrate via PMMA-assisted transfer strategies. Different from conventional acidic or alkaline solutions as etchant, deionized water was utilized here for the hydrophobicity/hydrophilicity property of the as-grown MoS_2/glass stack. The modified PMMA-assisted transfer process is as follows: Firstly, PMMA A4 was spin-coated on fresh MoS_2/glass stack at a speed of 500 r/min for 60 s, followed by 120 °C bake for 5 min. Secondly, the PMMA/MoS_2/glass stack was inclined gently inside a beaker with fresh deionized water. In this way, the PMMA/MoS_2 film can be delaminated from the glass substrate with the water penetrated into the interface between MoS_2 film and glass. Thirdly, cleaned HfLaO/Si or PI substrate was utilized here for the supporting substrate for the floating PMMA/MoS_2 films, followed by nitrogen blow dry and hot plate baking for stronger adhesion. Then, PMMA/MoS_2/HfLaO/Si or PI stack was immersed into acetone to remove PMMA, followed by IPA wash and nitrogen blow dry. Moreover, for the sample on rigid substrate, 350 °C annealing in argon atmosphere for 5 hours was carried out to remove organic residual.

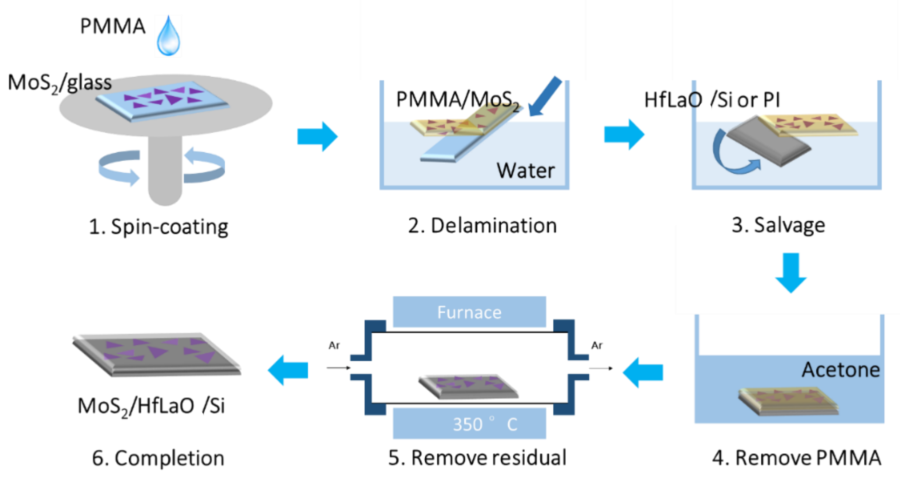

Supplementary Figure 8. Bilayer MoS_2 transfer process.

Although mechanically exfoliation strategy is accessible and easy to obtain large amount MoS_2 flake on target substrate, but the uncontrollable layer thickness, random location distribution and domain size hinder the device uniformity and future large area applications[10, 11]. On the other hand, CVD growth method possesses the advantages of layer number control[12], larger domain[13] and wafer-scale growth[14, 15] by adjusting substrate geometry, growth temperature, pressure, carrier gas and etc., overcoming the obstacle of mechanical exfoliation confronted. Moreover, with the

optimized synthesis, transfer and device fabrication, CVD growth monolayer or bilayer MoS_2 have higher potential of obtaining higher mobility and device performance than mechanical exfoliation ones[8, 16].

Supplementary Note 6. HfLaO growth and characterization.

Atomic layer deposition (ALD) was utilized here for deposition HfLaO film in this work. Before deposition, the substrate was cleaned by standard RCA cleaning process and a diluted buffed oxide etch (BOE) dip to remove organic and metallic contaminants, particles and unintentional oxides, followed by a deionized water rinse and drying. The substrate was then transferred to an ALD chamber to deposit HfLaO film at 300 °C, using TEMAHf, $La((^{i}Pr_2N)_2CH)_3$ and O_3 as the Hf precursor, La precursor, and oxygen source, respectively. The HfLaO film was achieved by controlling the HfO_2:La_2O_3 cycle ratio of 8:1. The rapid thermal annealing process was then performed in nitrogen ambient for 30 s at 500 °C. The corresponding CV and XPS characterizations are shown in Supplementary Figure 9 and Supplementary Figure 10. Dielectric constant of 26.7, La : Hf stoichiometric ratio of 0.06 : 1, and 0.14 portion of oxygen vacancy are obtained. 70 nm hafnium lanthanum oxide (HfLaO) was deposited as dielectric insulator on doped silicon substrates by ALD for MOSCAP fabrication. C-V measurement was performed at varied frequency from 50 - 1000 kHz, and oxide capacitance C_{ox} was extracted at 1000 kHz equal to 0.34 μFcm^{-2}. According to the formula of parallel plate capacitance, relative dielectric constant $\kappa = \varepsilon/\varepsilon_o = C_{ox}\, t_{ox} / \varepsilon_o = 26.7$, where ε represents dielectric constant, ε_o represents vacuum dielectric constant, and t_{ox} represents oxide thickness.

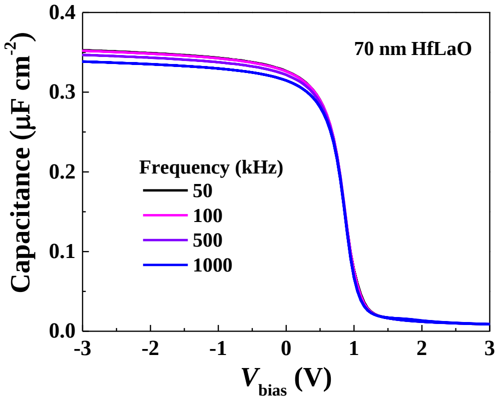

Supplementary Figure 9. MOSCAP C-V measurement results.

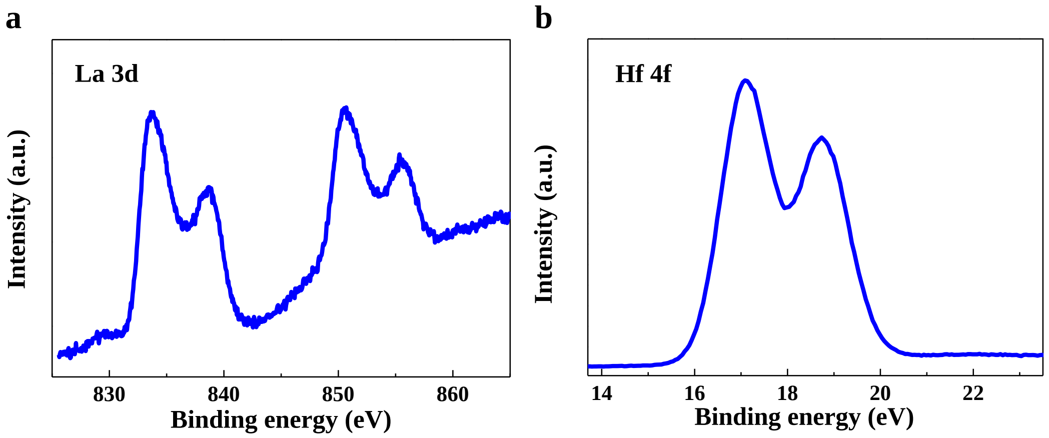

Supplementary Figure 10. XPS spectra of the **a,** La 3d, **b,** Hf 4f in the La:HfO_x film. The stoichiometric ratio of La: Hf is 0.06: 1. According to the XPS analysis, La:HfO_x can be represented as La:$HfO_{1.95}$, with 0.14 portion of oxygen vacancy.

Supplementary Note 7. Comparisons of Raman spectra and DC results for bilayer MoS_2 on SiO_2/Si and HfLaO/Si substrates.

As shown in the Raman spectra and PL in Supplementary Figure 11, there are two shifts in the E_{2g} and A_{1g} mode between the as-grown and transferred CVD MoS_2 bilayer, due to the strain stress release[17]. Moreover, A_{1g} peak redshift and FWMH broadening in Raman and A exciton redshift in PL spectrum are observed on HfLaO/Si substrate, indicating an interfacial oxygen vacancy doping of the MoS_2[18].

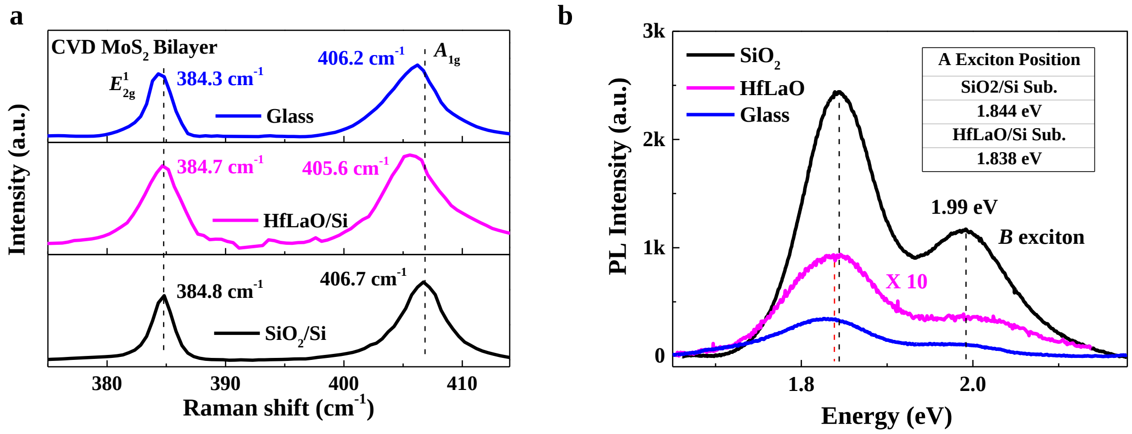

Supplementary Figure 11. **a,** Raman and **b,** PL spectrum of CVD MoS_2 bilayer as-grown on molten glass, transferred onto SiO_2/Si and HfLaO/Si substrates.

Bilayer MoS_2 devices on 90 nm SiO_2/Si and 70 nm HfLaO/Si were fabricated with the same experimental process side by side. Equivalent oxide thickness (EOT) of 70 nm HfLaO equals to 10.5 nm. In order to make a fair comparison for different back gate devices, V_{gs}^* is defined as $V_{gs} \cdot \frac{t_{ox}}{\mathrm{EOT}}$, where V_{gs} remain unchanged for SiO_2/Si substrate, but V_{gs} multiply 8.57 for HfLaO/Si substrate, as shown in Supplementary Figure 12a. We can find a negative V_T shift about -12.4 V on HfLaO/Si substrate, compared with SiO_2/Si, indicating a charge transfer and positive fixed charge[19]. Compared with 0.44 portion oxygen vacancy and 0.1 portion excess oxgen in HfO_x, our 0.14 portion oxygen vacancy in La:HfO_x induce a moderate V_T shift than in literature[18], indicating a consistent interfacial oxygen vacancy doping. MoS_2 on HfLaO/Si substrate exhibit a higher ON current and lower contact resistance than on SiO_2/Si, as depicted in Supplementary Figure 12b.

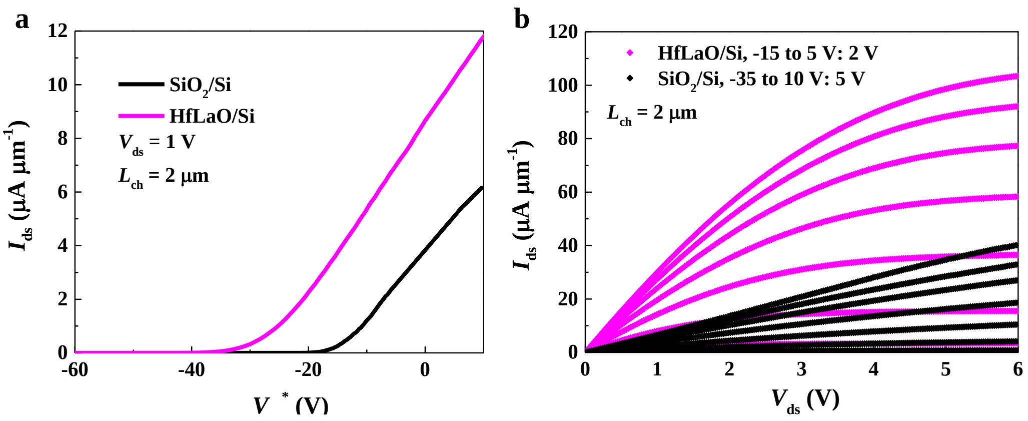

Supplementary Figure 12. **a,** Transfer and **b,** Output characteristics curves of MoS_2 bilayer device on SiO_2/Si and HfLaO/Si substrate.

Supplementary Note 8. Extraction of intrinsic mobility

Field-effect mobility is firstly extracted from the transfer characteristics in the linear region using the equation

$\mu_{FE} = g_m L_{ch} / W C_{ox} V_{ds}$ (Supplementary Equations 6)

where g_m is the peak trans-conductance, L_{ch} and W are the channel length and width, respectively, C_{ox} is the back gate capacitance, and V_{ds} is the drain-to-source voltage. Then, to eliminate the parasitic effects from contact resistance, the effective V_{gs}, V_{ds}, and intrinsic transconductance are given by $V_{gs}' = V_{gs} - I_{ds}R_c$, $V_{ds}' = V_{ds} - 2I_{ds}R_c$, and $g_m' \approx g_m/(1 - R_c\, g_m)$, respectively, where R_C is the

contact resistance extracted by the transfer length method[20, 21]. Supplementary Table 1 compares the mobility and domain size values with previous reported results.

Supplementary Table 1. Comparison of CVD growth bilayer MoS_2

Ref	**Substrates**	**Domain Size (μm)**	**μ ($cm^2V^{-1}s^{-1}$)**
8	SiO_2/Si	21	22*
9	Mica, fused silica, SiO_2/Si	12	--
22	SiO_2/Si	12.3	4.7
23	SiO_2/Si	6.8	--
24	MoO_2	22	0.3
25	SiO_2/Si	60	--
This work	Molten glass	200	36<br>127 (4.3 K)

Notes: a) Domain sizes were measured and calculated according to the scale bars in the references.
b) * Statistics of electron mobility of 1-3 layers MoS_2

Supplementary Note 9. Discussion of the mobility temperature dependence at 4.3 K

The intrinsic mobility can be fitted using the following function:

$1/\mu(T) = 1/\mu_{imp} + 1/\mu_{ph}(T)$ (Supplementary Equations 7)

Where μ_{imp} represents the contribution from Coulomb impurity scattering and μ_{ph} is the temperature-dependent contribution from phonon scattering. Moreover, the fitted $\mu_{ph}(T)$ is well described by a power law ($\mu_{ph} \sim T^{-\gamma}$) above 100 K. This behavior is consistent with mobility limited MoS_2 optical phonons, which is theoretically predicted to have an exponent of ~1.69 in monolayer [26] and ~2.5 in bulk [27] MoS_2 at T > 100 K. A temperature dependence was shown in Supplementary Figure 13, with the exponent γ equal to 1.48, consistent with literatures [28]. The bilayer mobility begins to saturate below 100 K, a temperature by which scattering from optical phonons is expected

to become negligible[29] and long-range Coulomb impurity scattering becomes dominant. High-κ gate dielectric used as the top-gate dielectrics[30] or defects and interface decoration[31] can further effectively screen phonon and Coulomb impurity scattering as shown in Supplementary Table 2. In short, the mobility increase from 36 $cm^2V^{-1}s^{-1}$ at 300 K to 127 $cm^2\ V^{-1}s^{-1}$ at 4.3 K due to the reduced phonon scattering.

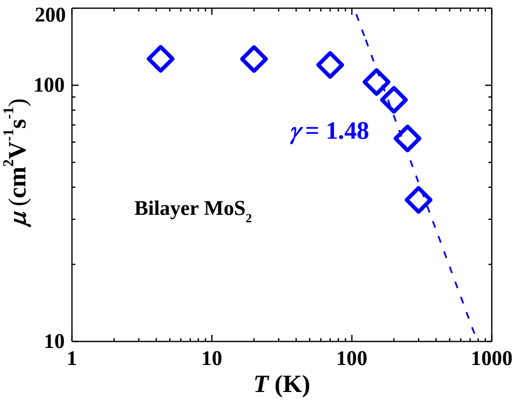

Supplementary Figure 13. The extracted carrier mobility vs. temperatures.

Supplementary Table 2. Comparison of γ value from other groups

Ref	**Layer number**	**Process**	**γ value**	**Method**
28	Bilayer	Exfoliation	2.5	hBN-encapsulation
29	Monolayer	CVD	0.7	Two-step annealing
30	Monolayer	Exfoliation	0.55-0.78	HfO_2 encapsulation
31	Monolayer	Exfoliation	0.72	DS-treated
32	Bilayer	Exfoliation	1.1	Vacuum annealing
This work	Bilayer	CVD	1.48	HfLaO back-gate dielectric

Supplementary Note 10. Scaling behavior and contact resistance

Supplementary Figure 14 shows the channel length dependent output current. The slight discrepancy of drain current from I_{on} - L_{ch}^{-1} relationship with channel length scaling down is shown. The discrepancy is attributed to two reasons. One reason is the contact resistance which does not scale with channel length where the extracted contact resistance can be seen in Supplementary Figure 15 using a transfer-length method. The second reason is that the apparent mobility decreases at shorter channel lengths.

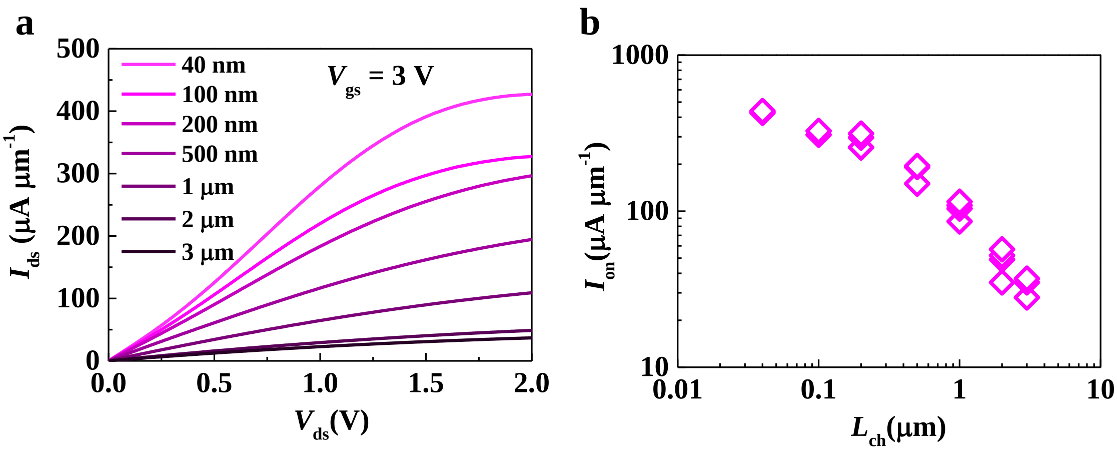

Supplementary Figure 14. **a,** Output curves at the same 3 V gate bias from a series of transistors with different channel lengths at room temperature. **b,** Maximum on-state current at V_{gs} = 3 V and V_{ds} = 2 V for 23 devices with 7 different channel lengths.

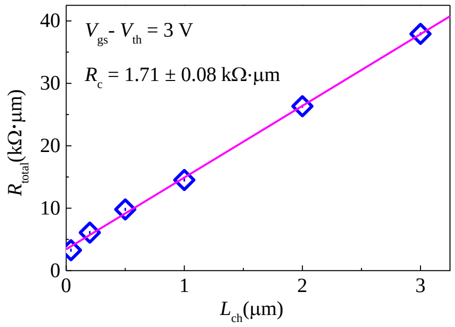

Supplementary Figure 15. Total device resistance R_{total} vs. L_{ch} measured by TLM, at V_{gs}- V_{th} = 3 V. The good linear fits to the total device resistance normalized by width (R_{total}) vs. L_{ch}, demonstrating uniform contacts. The vertical intercept of the linear fit yields the total contact resistance ($2R_c$). The Ni/Au contacts for bilayer MoS_2 used here obtain R_c of about 1.7 kΩ·μm.

Supplementary Note 11. DC characterization bilayer MoS_2 RF transistors

Supplementary Figure 16 shows the DC characteristics of the RF transistors. It should be noted that, the typical drain-source conductance is smaller than 10 μS μm^{-1} in the region of operation for RF measurement. And, the corresponding output resistance, r_o, is larger than 0.1 MΩ·μm for the devices with gate lengths from 300 nm to 90 nm.

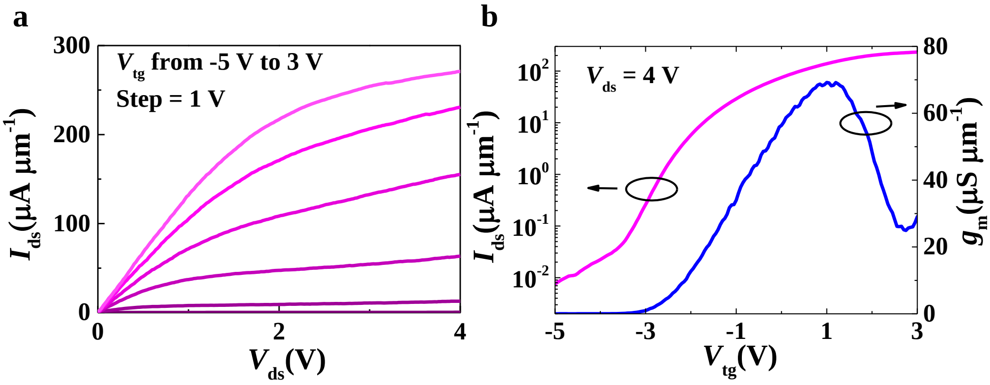

Supplementary Figure 16. DC characterizations of CVD bilayer MoS_2 RF transistors. **a,** I_{ds}-V_{ds} output characteristics of an RF transistor with L_g = 190 nm. The V_{ds} is swept from 0 to 4 V with different V_{tg}. The V_{tg} is swept from −5 to 3 V in step of 1 V. A current density of 270 μA μm^{-1} is achieved at V_{tg}= 3 V and V_{ds} = 4 V. **b,** The I_{ds}-V_{tg} transfer characteristics and the corresponding g_m-V_{tg} trans-conductance curves of the same devices at V_{ds} = 4 V. The peak g_m is 70 μS μm^{-1} biased at V_{gs} = 1 V and V_{ds} = 4 V.

Supplementary Note 12. Discussion on the improvement of f_T and f_{max}

The cut-off frequency (f_T), defined as the frequency at which the current gain becomes unity, is one of the most important figures-of-merit for evaluating the performance of RF devices. In a well-behaved field-effect transistor, the intrinsic cut-off frequency can be related to trans-conductance g_m by the following equation:

$$f_T = \frac{g_m}{2\pi C_g} \quad \text{(Supplementary Equations 8)}$$

where C_g is the gate capacitance. And, actually trans-conductance g_m is associated with carrier mobility μ, gate voltage V_{gs}, threshold voltage V_{th} and gate length L_g. Thus, for the devices with long gate length:

$$f_T \approx \frac{\mu(V_g - V_T)}{2\pi L_g^{\ 2}}$$ (Supplementary Equations 9)

and for the devices with short gate length:

$$f_T \approx \frac{\upsilon_{sat}}{2\pi L_g}$$ (Supplementary Equations 10)

Also, a major limitation on the performance of MoS_2 for RF applications arises from the high values of contact resistance R_c determining the source and drain parasitic resistances[33]. The lower contact resistance result from higher density of states and carrier mobility of CVD bilayer MoS_2 when compared with monolayer one. Thus, high carrier mobility, low contact resistance and short gate lengths can lead to higher f_T of MoS_2 transistors. Maximum oscillation frequency, f_{max}, which is defined as the frequency at which the power gain equals to unity. f_{max} can be calculated as

$$f_{\max} = \frac{f_T}{2\sqrt{g_{ds}(R_g + R_s) + 2\pi f_T R_g C_{gd}}}$$ (Supplementary Equations 11)

where g_{ds} is the output conductance, R_g is the gate resistance, R_s is the source resistance, and C_{gd} is gate-to-drain capacitance[34]. The typical drain-source conductance $g_{ds} = \partial I_{ds} / \partial V_{ds}$ are smaller than 10 μSμm^{-1} in the region of operation for RF measurement. And, the corresponding r_o ($r_o = 1/g_{ds} = \partial V_{ds} / \partial I_{ds}$) are larger than 0.1 MΩ·μm. As a result, further improvement of f_{max} can be obtained by higher f_T, high output resistance and reduction of gate resistance and other parasitic resistances by device structure optimizations.

Supplementary Note 13. Gummel's method.

Supplementary Figure 17 shows the Gummel's method for the cut-off frequency.

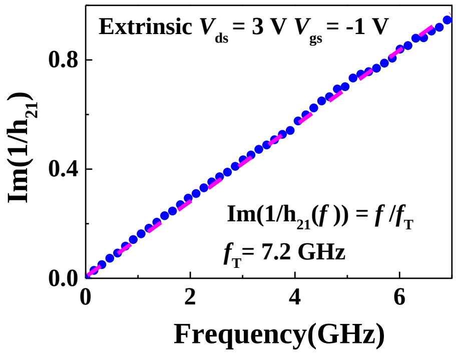

Supplementary Figure 17. The imaginary part of $1/h_{21}$ as a function of frequency. The resulting in f_T value is in agreement with the presented in the main manuscript.

Supplementary Note 14. De-embedded f_T

The de-embedded f_T give an idea of the intrinsic material quality, as well as the parasitic effect of the device geometry and composition. The de-embedding procedure performed in 2D RF papers use various open structures and results in intrinsic de-embedding and standard de-embedding[35, 36]. As shown in Supplementary Figure 18, intrinsic de-embedded f_T of 78 GHz and f_{max} of 34 GHz are obtained for the best devices data. Intrinsic de-embedding process not only removes the electrode pads but also metal interconnects (and their associated capacitances) in the device structure. Here, we would like to emphasize that $f_{T,int}$ only represents the upper limit of the possible frequency for this transistor. In any practical applications, the C_{gs} and C_{gd} of the device always significantly affect the device performance. Supplementary Table 3 compares the RF performance of previously reported 2D semiconductors including MoS_2 and black phosphorus.

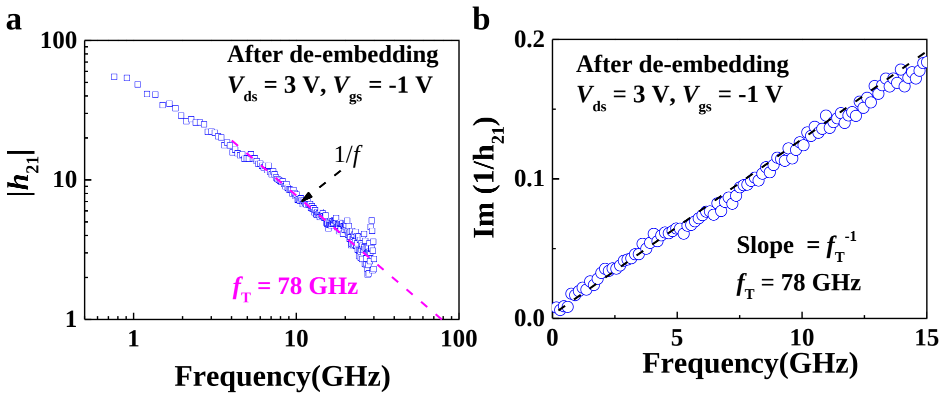

Supplementary Figure 18. RF characterizations of CVD bilayer MoS_2 RF transistors after intrinsic de-embedding. **a**,. Short-circuit current gain $|h_{21}|$ of the 90 nm channel length device after intrinsic de-embedding. **b,** Linear fitting using Gummel's method.

Supplementary Table 3. Comparison of extrinsic RF performance based on 2D semiconductors

Ref	Materials	Substrates	L_g (nm)	$f_{T,extrinsic}$ (GHz)	$f_{max,extrinsic}$ (GHz)	f_{max}/f_T	$f_{T,intrinsic}$ (GHz)	v_{sat} ($\times$ 10^6 cm/s)
37	Exfoliated MoS_2	SiO_2/Si	68	1.3	1.5	1.2	42	1.8
		Quartz		10.2	14.5	1.4	--	--
		PI		4.7	5.4	1.1	13.5	0.58
38	Exfoliated MoS_2	SiO_2/Si	240	2.1	--	--	6	0.9
39	Exfoliated MoS_2	SiO_2/Si	70	6	--	--	25	1.1
36	Exfoliated BP	SiO_2/Si	300	8	12	1.5	51	9.6
40	Exfoliated BP	PI	250	7	10.3	1.5	17.5	5.5
41	CVD Monolayer MoS_2	SiO_2/Si	250	2.8	3.6	1.3	6.7	1.05
42	CVD Monolayer MoS_2	PI	500	2.7	2.1	0.8	5.6	1.76
43	CVD Monolayer MoS_2	SiO_2/Si	150	3.3	9.8	3	20	1.88
This Work	CVD Bilayer MoS_2	HfLaO/Si	90	7.2	23	3.2	78	4.4
		PI	300	4	9	2.3	4.4	0.83

Supplementary Note 15. Extrinsic RF characterization of devices with gate lengths of 190 and 300 nm.

Supplementary Figure 19 shows the cut-off frequencies and maximum oscillation frequencies of the longer channel devices of 190 nm and 300 nm.

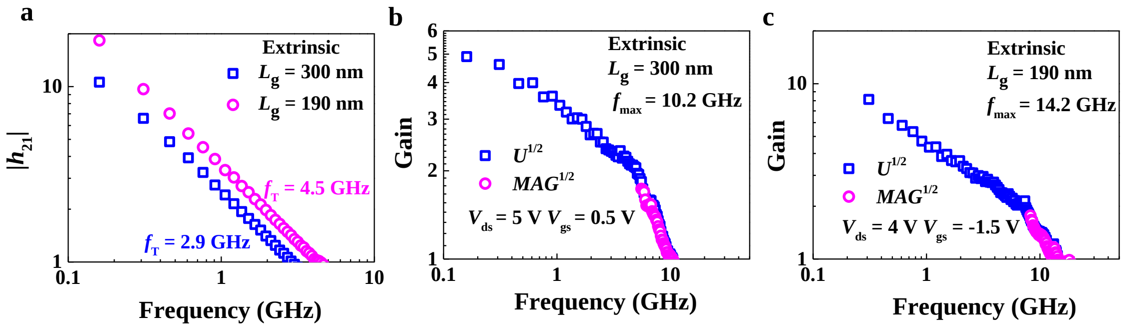

Supplementary Figure 19. Extrinsic RF characterizations of CVD bilayer MoS_2 RF transistors with gate lengths of 190 nm and 300 nm. **a,** Short-circuit current gain $|h_{21}|$ of devices with gate lengths of 300 nm and 190 nm. The extrinsic cut-off frequencies f_T are 2.9 and 4.5 GHz, respectively. Where V_{ds}= 5 V and V_{gs}= 0.5 V for the device with gate length of 300 nm, and V_{ds}= 4 V and V_{gs}=0 V for the device with gate length of 160 nm. **b,** Unilateral power gain U , and maximum available gain MAG versus frequency for a device with gate length of 300 nm. The extrinsic maximum oscillation frequency f_{max} is 10.2 GHz. **c,** Unilateral power gain U, and maximum available gain MAG versus frequency for a device with gate length of 190 nm. The extrinsic f_{max} is 14.2 GHz.

Supplementary Note 16. I_{ds}-V_{gs} and I_{ds}-V_{ds} plot for bilayer MoS_2 on flexible substrates

Supplementary Figure 20 shows the I_{ds}-V_{gs} and I_{ds}-V_{ds} plots of a device with L_g = 600 nm for bilayer MoS_2 on flexible substrates. On/off current ratio of about 10^8 and on current density of 40 $\mu A \mu m^{-1}$ were achieved. For the device with L_g = 300 nm, On/off current ratio of about 10^8 and higher current density of 70 $\mu A \mu m^{-1}$ was obtained. The degradation of device DC performance is often found in literatures while converting the substrate from rigid to flexible substrate with a similar fabrication process[37, 42]. Which can be attributed to substrate roughness and poor thermal conductivity that could partly degrade the charge transport properties in these atomically thin materials[44].

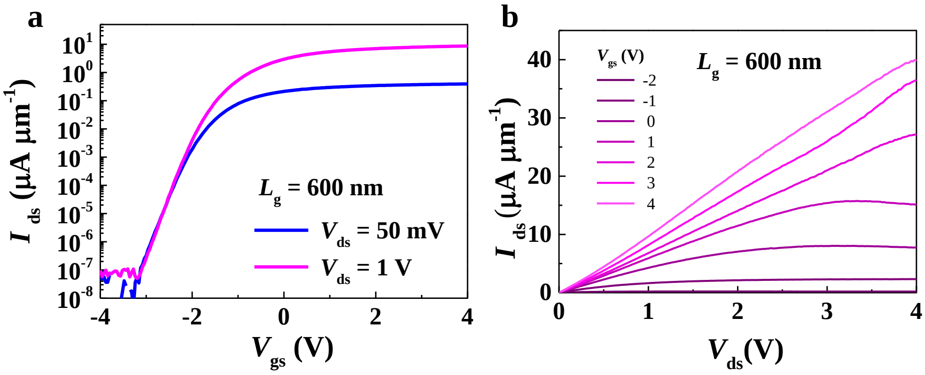

Supplementary Figure 20. **a,** The transfer characteristics of MoS_2 FET on PI. The switching ration (I_{on}/I_{off}) is about 10^8. **b,** Output curves, I_{ds}-V_{ds}, for the same device. The device reach a maximum current density of 40 μA μm^{-1} at a V_{ds} of 4 V, V_{gs} of 4 V.

Supplementary Note 17. DC and RF performance of flexible MoS_2 transistors under various bending conditions.

Supplementary Figure 21 shows the device performance of the flexible transistors at bending conditions.

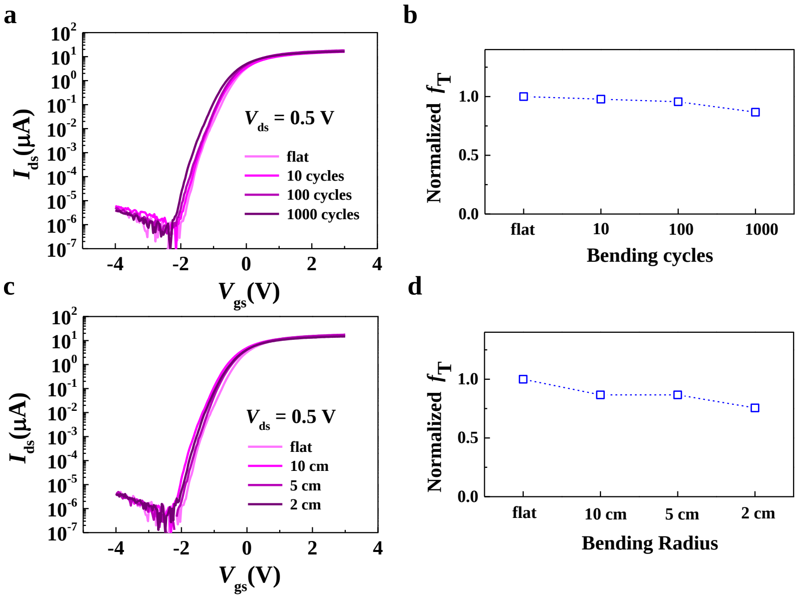

Supplementary Figure 21. **a,** and **b,** display the $I_{ds}V_{gs}$ and normalized f_T of the flexible bilayer MoS_2 transistors after different bending cycles with the bending radius keep at 10 cm. **c,** and **d,** display the $I_{ds}V_{gs}$ and normalized f_T after four different bending conditions: flat, R= 10 cm 1000 cycles, R= 5 cm 1000 cycles, R= 2 cm 1000 cycles.

Supplementary Note 18. IF gain vs. frequency.

Supplementary Figure 22 shows the IF gain at different frequencies from 1 GHz to 1.7 GHz for rigid and flexible mixers.

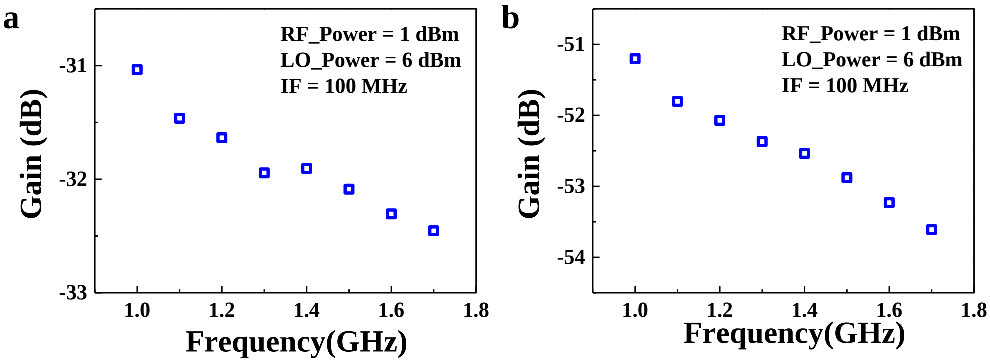

Supplementary Figure 22. **a,** and **b,** IF conversion gain vs. frequency for the rigid and flexible mixers.

References

1. Bhaviripudi, S., Jia, X., Dresselhaus, M.S. & Kong, J. Role of kinetic factors in chemical vapor deposition synthesis of uniform large area graphene using copper catalyst. *Nano Lett.* **10**, 4128-4133 (2010).
2. Zhou, S., Gan, L., Wang, D., Li, H. & Zhai, T. Space-confined vapor deposition synthesis of two dimensional materials. *Nano Res.* **11**, 2909-2931 (2018).
3. Pierson, H.O. Handbook of chemical vapor deposition: principles, technology and applications. (William Andrew, 1999).
4. Zhou, J., Lin, J., Huang, X., Zhou, Y., Chen, Y., Xia, J., Wang, H., Xie, Y., Yu, H., Lei, J., Wu, D., Liu, F., Fu, Q., Zeng, Q., Hsu, C.-H., Yang, C., Lu, L., Yu, T., Shen, Z., Lin, H., Yakobson, B.I., Liu, Q., Suenaga, K., Liu, G. & Liu, Z. A library of atomically thin metal chalcogenides. *Nature* **556**, 355-359 (2018).
5. Chen, J., Zhao, X., Tan, S.J.R., Xu, H., Wu, B., Liu, B., Fu, D., Fu, W., Geng, D., Liu, Y., Liu, W., Tang, W., Li, L., Zhou, W., Sum, T.C. & Loh, K.P. Chemical vapor deposition of large-size monolayer $MoSe_2$ crystals on molten glass. *J. Am. Chem. Soc.* **139**, 1073-1076 (2017).
6. Einax, M., Dieterich, W. & Maass, P. Colloquium: cluster growth on surfaces: densities, size

distributions, and morphologies. *Rev. Mod. Phys.* **85**, 921-939 (2013).

7. Yang, P., Zou, X., Zhang, Z., Hong, M., Shi, J., Chen, S., Shu, J., Zhao, L., Jiang, S., Zhou, X., Huan, Y., Xie, C., Gao, P., Chen, Q., Zhang, Q., Liu, Z. & Zhang, Y. Batch production of 6-inch uniform monolayer molybdenum disulfide catalyzed by sodium in glass. *Nat. Commun.* **9**, 979 (2018).
8. Zheng, J., Yan, X., Lu, Z., Qiu, H., Xu, G., Zhou, X., Wang, P., Pan, X., Liu, K. & Jiao, L. High-mobility multilayered MoS_2 flakes with low contact resistance grown by chemical vapor deposition. *Adv. Mater.* **29**, 1604540 (2017).
9. Liu, K., Zhang, L., Cao, T., Jin, C., Qiu, D., Zhou, Q., Zettl, A., Yang, P., Louie, S.G. & Wang, F. Evolution of interlayer coupling in twisted molybdenum disulfide bilayers. *Nat. Commun.* **5**, 4966 (2014).
10. Desai, S.B., Madhvapathy, S.R., Sachid, A.B., Llinas, J.P., Wang, Q., Ahn, G.H., Pitner, G., Kim, M.J., Bokor, J., Hu, C., Wong, H.S.P. & Javey, A. MoS_2 transistors with 1-nanometer gate lengths. *Science* **354**, 99 (2016).
11. Radisavljevic, B., Radenovic, A., Brivio, J., Giacometti, V. & Kis, A. Single-layer MoS_2 transistors. *Nat. Nanotechnol.* **6**, 147 (2011).
12. Lee, Y.-H., Zhang, X.-Q., Zhang, W., Chang, M.-T., Lin, C.-T., Chang, K.-D., Yu, Y.-C., Wang, J.T.-W., Chang, C.-S., Li, L.-J. & Lin, T.-W. Synthesis of large-area MoS_2 atomic layers with chemical vapor deposition. *Adv. Mater.* **24**, 2320-2325 (2012).
13. Chen, W., Zhao, J., Zhang, J., Gu, L., Yang, Z., Li, X., Yu, H., Zhu, X., Yang, R., Shi, D., Lin, X., Guo, J., Bai, X. & Zhang, G. Oxygen-assisted chemical vapor deposition growth of large single-crystal and high-quality monolayer MoS_2. *J. Am. Chem. Soc.* **137**, 15632-15635 (2015).
14. Kang, K., Xie, S., Huang, L., Han, Y., Huang, P.Y., Mak, K.F., Kim, C.-J., Muller, D. & Park, J. High-mobility three-atom-thick semiconducting films with wafer-scale homogeneity. *Nature* **520**, 656 (2015).
15. Yu, H., Liao, M., Zhao, W., Liu, G., Zhou, X.J., Wei, Z., Xu, X., Liu, K., Hu, Z., Deng, K., Zhou, S., Shi, J.-A., Gu, L., Shen, C., Zhang, T., Du, L., Xie, L., Zhu, J., Chen, W., Yang, R., Shi, D. & Zhang, G. Wafer-scale growth and transfer of highly-oriented monolayer MoS_2 continuous films. *ACS Nano* **11**, 12001-12007 (2017).
16. Dumcenco, D., Ovchinnikov, D., Marinov, K., Lazić, P., Gibertini, M., Marzari, N., Sanchez,

O.L., Kung, Y.-C., Krasnozhon, D., Chen, M.-W., Bertolazzi, S., Gillet, P., Fontcuberta i Morral, A., Radenovic, A. & Kis, A. Large-area epitaxial monolayer MoS_2. *ACS Nano* **9**, 4611-4620 (2015).

17. Liu, Z., Amani, M., Najmaei, S., Xu, Q., Zou, X., Zhou, W., Yu, T., Qiu, C., Birdwell, A.G., Crowne, F.J., Vajtai, R., Yakobson, B.I., Xia, Z., Dubey, M., Ajayan, P.M. & Lou, J. Strain and structure heterogeneity in MoS_2 atomic layers grown by chemical vapour deposition. *Nat. Commun.* **5**, 5246 (2014).
18. Rai, A., Valsaraj, A., Movva, H.C.P., Roy, A., Tutuc, E., Register, L.F. & Banerjee, S.K. Interfacial-oxygen-vacancy mediated doping of MoS_2 by high-κ dielectrics. *73rd Annual Device Research Conference (DRC)*, 189-190 (2015).
19. McClellan, C.J., Yalon, E., Smithe, K.K.H., Suryavanshi, S.V. & Pop, E. Effective n-type doping of monolayer MoS_2. *2017 75th Annual Device Research Conference (DRC)*, 1-2 (2017).
20. Chou, S.Y. & Antoniadis, D.A. Relationship between measured and intrinsic transconductances of FET's. *IEEE Trans. Electron Devices* **34**, 448-450 (1987).
21. Sze, S.M. & Ng, K.K. Physics of semiconductor devices. (John wiley & sons, 2006).
22. Zobel, A., Boson, A., Wilson, P.M., Muratov, D.S., Kuznetsov, D.V. & Sinitskii, A. Chemical vapour deposition and characterization of uniform bilayer and trilayer MoS_2 crystals. *J. Mater. Chem. C* **4**, 11081-11087 (2016).
23. Hao, S., Yang, B. & Gao, Y. Controllable growth and electrostatic properties of Bernal stacked bilayer MoS_2. *J. Appl. Phys.* **120**, 124310 (2016).
24. Wang, X., Feng, H., Wu, Y. & Jiao, L. Controlled synthesis of highly crystalline MoS_2 flakes by chemical vapor deposition. *J. Am. Chem. Soc.* **135**, 5304-5307 (2013).
25. Ye, H., Zhou, J., Er, D., Price, C.C., Yu, Z., Liu, Y., Lowengrub, J., Lou, J., Liu, Z. & Shenoy, V.B. Toward a mechanistic understanding of vertical growth of van der Waals stacked 2D materials: a multiscale model and experiments. *ACS Nano* **11**, 12780-12788 (2017).
26. Kaasbjerg, K., Thygesen, K.S. & Jacobsen, K.W. Phonon-limited mobility in n-type single-layer MoS_2 from first principles. *Phys. Rev. B* **85**, 115317 (2012).
27. Fivaz, R. & Mooser, E. Mobility of charge carriers in semiconducting layer structures. *Phys. Rev.* **163**, 743-755 (1967).

28. Cui, X., Lee, G.-H., Kim, Y.D., Arefe, G., Huang, P.Y., Lee, C.-H., Chenet, D.A., Zhang, X., Wang, L., Ye, F., Pizzocchero, F., Jessen, B.S., Watanabe, K., Taniguchi, T., Muller, D.A., Low, T., Kim, P. & Hone, J. Multi-terminal transport measurements of MoS_2 using a van der Waals heterostructure device platform. *Nat. Nanotechnol.* **10**, 534 (2015).
29. Schmidt, H., Wang, S., Chu, L., Toh, M., Kumar, R., Zhao, W., Castro Neto, A.H., Martin, J., Adam, S., Özyilmaz, B. & Eda, G. Transport properties of monolayer MoS_2 grown by chemical vapor deposition. *Nano Lett.* **14**, 1909-1913 (2014).
30. Radisavljevic, B. & Kis, A. Mobility engineering and a metal–insulator transition in monolayer MoS_2. *Nat. Mater.* **12**, 815 (2013).
31. Yu, Z., Pan, Y., Shen, Y., Wang, Z., Ong, Z.-Y., Xu, T., Xin, R., Pan, L., Wang, B., Sun, L., Wang, J., Zhang, G., Zhang, Y.W., Shi, Y. & Wang, X. Towards intrinsic charge transport in monolayer molybdenum disulfide by defect and interface engineering. *Nat. Commun.* **5**, 5290 (2014).
32. Baugher, B.W.H., Churchill, H.O.H., Yang, Y. & Jarillo-Herrero, P. Intrinsic electronic transport properties of high-quality monolayer and bilayer MoS_2. *Nano Lett.* **13**, 4212-4216 (2013).
33. Holland, K.D., Alam, A.U., Paydavosi, N., Wong, M., Rogers, C.M., Rizwan, S., Kienle, D. & Vaidyanathan, M. Impact of contact resistance on the f_T and f_{max} of graphene versus MoS_2 transistors. *IEEE Trans. Nanotechnol.* **16**, 94-106 (2017).
34. Han, S.J., Oida, S., Jenkins, K.A., Lu, D. & Zhu, Y. Multifinger embedded T-shaped gate graphene RF transistors with high f_{max}/f_T ratio. *IEEE Electr. Dev. Lett.* **34**, 1340-1342 (2013).
35. Petrone, N., Meric, I., Chari, T., Shepard, K.L. & Hone, J. Graphene field-effect transistors for radio-frequency flexible electronics. *IEEE J. Electr. Dev. Soc.* **3**, 44-48 (2014).
36. Wang, H., Wang, X., Xia, F., Wang, L., Jiang, H., Xia, Q., Chin, M.L., Dubey, M. & Han, S.-j. Black phosphorus radio-frequency transistors. *Nano Lett.* **14**, 6424-6429 (2014).
37. Cheng, R., Jiang, S., Chen, Y., Liu, Y., Weiss, N., Cheng, H.-C., Wu, H., Huang, Y. & Duan, X. Few-layer molybdenum disulfide transistors and circuits for high-speed flexible electronics. *Nat. Commun.* **5**, 5143 (2014).
38. Krasnozhon, D., Lembke, D., Nyffeler, C., Leblebici, Y. & Kis, A. MoS_2 transistors operating at gigahertz frequencies. *Nano Lett.* **14**, 5905-5911 (2014).

39. Krasnozhon, D., Dutta, S., Nyffeler, C., Leblebici, Y. & Kis, A. High-frequency, scaled MoS_2 transistors. *2015 IEEE International Electron Devices Meeting (IEDM)*, 27.24.21-27.24.24 (2015).

40. Zhu, W., Park, S., Yogeesh, M.N., McNicholas, K.M., Bank, S.R. & Akinwande, D. Black phosphorus flexible thin film transistors at gighertz frequencies. *Nano Lett.* **16**, 2301-2306 (2016).

41. Sanne, A., Ghosh, R., Rai, A., Yogeesh, M.N., Shin, S.H., Sharma, A., Jarvis, K., Mathew, L., Rao, R., Akinwande, D. & Banerjee, S. Radio frequency transistors and circuits based on CVD MoS_2. *Nano Lett.* **15**, 5039-5045 (2015).

42. Chang, H.-Y., Yogeesh, M.N., Ghosh, R., Rai, A., Sanne, A., Yang, S., Lu, N., Banerjee, S.K. & Akinwande, D. Large-area monolayer MoS_2 for flexible low-power RF nanoelectronics in the GHz regime. *Adv. Mater.* **28**, 1818-1823 (2016).

43. Sanne, A., Park, S., Ghosh, R., Yogeesh, M.N., Liu, C., Mathew, L., Rao, R., Akinwande, D. & Banerjee, S.K. Embedded gate CVD MoS_2 microwave FETs. *npj 2D Mater. Appl.* **1**, 26 (2017).

44. Park, S., Shin, S.H., Yogeesh, M.N., Lee, A.L., Rahimi, S. & Akinwande, D. Extremely high-frequency flexible graphene thin-film transistors. *IEEE Electr. Dev. Lett.* **37**, 512-515 (2016).
